# Gender-dimorphic regulation of DJ1 and its interactions with metabolic proteins in streptozotocin-induced diabetic rats

**DOI:** 10.1111/jcmm.12490

**Published:** 2015-02-27

**Authors:** Harmesh N Chaudhari, Sang Woo Kim, Jong Won Yun

**Affiliations:** Department of Biotechnology, Daegu UniversityKyungbuk, Korea

**Keywords:** DJ1, gender differences, GeneMANIA, streptozotocin, stress proteins, type 1 diabetes mellitus

## Abstract

Regulation of DJ1 is associated with a number of human diseases. To determine the involvement of DJ1 in progression of diabetes in a gender-dependent manner, we investigated its tissue-specific expression in streptozotocin (STZ)-induced diabetic male and female rats in this study. In animal experiments, females showed greater susceptibility towards developing diabetes because of lower insulin secretion and higher blood glucose levels as compared to male diabetic rats upon exposure to STZ. Immunoblotting confirmed sexually dimorphic regulation of DJ1 in various metabolic tissues such as the liver, pancreas and skeletal muscle. Immunofluorescence analysis revealed the location as well as reinforced the gender-dependent expression of DJ1 in hepatic tissue. Co-immunoprecipitation assay identified several interacting proteins with DJ1 whose functions were shown to be involved in various metabolic pathways *viz*. antioxidative and stress defence system, protein and methionine metabolism, nitrogen metabolism, urea metabolism, *etc*. Using GeneMANIA, a predictive web interface for gene functions, we showed for the first time that DJ1 may regulate T1DM *via* the JNK1 pathway, suggesting DJ1 interacts with other proteins from various metabolic pathways. We anticipate that the current data will provide insights into the aetiology of T1DM.

## Introduction

Type 1 diabetes mellitus (T1DM) is an autoimmune disease wherein an improper autoimmune response affects and destroys insulin-producing β-cells in pancreatic islets, which leads to impaired blood glucose levels 1. Impairment of insulin biosynthesis and secretion are characteristic of persistent hyperglycemia, leading to insulin resistance 2,3 coupled with oxidative stress 4 and inflammation 5. Diabetes affects metabolism in various metabolic tissues *viz*. the pancreas, skeletal muscle, kidney, liver, *etc*. The liver is widely known as a metabolic centre and plays a vital role in several metabolic processes, including carbohydrate metabolism, detoxification of harmful substances, breakdown of insulin and other hormones, and storage of numerous metabolic substances 6.

As gender development and reproduction have different metabolic requirements, hepatic tissue displays significant sexual dimorphism along with protein content 7. Gender-dimorphic oxidative metabolism has been reported in females showing elevated levels of protein content, phosphorylative capacity, and oxidative capacity in rat liver mitochondria, and energized levels of mitochondria have been observed in females as compared to male counterparts 8. Various studies have attempted to tackle the issue of T1DM 9–11. A recent study in our laboratory demonstrated gender dimorphism in terms of hepatic protein expression associated with T1DM in rats 12.

DJ1 is a ubiquitous, cytoprotective, multifunctional and highly conserved protein in mammals 13,14. It is associated with Parkinson's disease 15 and protects neurons from mitochondrial and oxidative stress 16. It is recognized that loss of DJ1 increases ROS production 17. Aside from its roles in oxidative stress and neurodegenerative disease, DJ1 has gained attention for its involvement in metabolic complications. For example, DJ1 is known to participate in pancreatic β-cell regulation and protection 18 as well as prevention of nitric oxide-mediated apoptosis 19. Until now, there are conflicting results concerning DJ1 expression in metabolic diseases. For instance, overexpression of DJ1 has been observed in mice exposed to a high-fat diet (HFD) 18, whereas decreased expression of DJ1 has been detected in the islets of elderly T2DM patients in a gender-dependent manner 14. In contrast, elevated expression of DJ1 was previously found in pancreatic islets of females as compared to their age-matched male counterparts 14, and a similar expression pattern of DJ1 was noted in heart tissue of streptozotocin (STZ)-induced male Sprague-Dawley (SD) rats 20. Taken together, these reports show that DJ1 expression is up-regulated in rats and mice fed a HFD, whereas it is down-regulated in STZ-induced rats as well as in T2DM human pancreatic islets. Therefore, it can be inferred that the presence of DJ1 protects against oxidative stress or diet-induced complications, whereas absence or reduced expression of DJ1 protects against diabetes-related complications.

To date, no evidence has linked DJ1 expression to diabetes pathophysiology. Therefore, the aim of this study was to compare the gender-dependent expression of DJ1 in various metabolic tissues as well as to identify the interacting partners of DJ1 in hepatic tissue. These interactions were further validated by immunoblotting and the predictive web interface GeneMANIA. To the best of our knowledge, this is the first study to elucidate the gender-dependent and tissue-specific expression of DJ1 along with its protein–protein interactions in the livers of STZ-induced rats.

## Materials and methods

### Animal experiment

Male and female SD rats at 11 weeks of age were purchased from Daehan Experiment Animals (Hanam, Korea) and housed individually in metabolic cages at a temperature of 23 ± 2°C and relative humidity of 55% in a controlled room with a 12 hrs light/dark cycle for 1 week in the animal facility of our laboratory. Diet composed of standard pelleted raw chow and water was made available *ad libitum* to animals. Rat feeds were purchased from Feed Korea Laboratory (Hanam, Korea). Male (*n* = 24) and female (*n* = 24) rats were randomly divided into two groups. After 1 week of adaptation, 48 animals were subdivided into four groups, *viz*. male control (*n* = 8); male STZ (*n* = 16); female control (*n* = 8); female STZ (*n* = 16). To induce diabetes, STZ dissolved in 0.01 M sodium citrate buffer (pH 4.5) was injected intravenously into STZ-treated males and females at a dose of 50 mg/kg body weight, whereas injection of vehicle was administered to control rats. Body weight and blood glucose levels were determined at the same time-point for the 2-week period. Glucose oxidase reagent strips and compact glucometer (Lifescan, Inc., Milpitas, CA, USA) were used to determine blood glucose levels, and rats with a blood glucose level over 300 mg/dl were confirmed as diabetic. Finally, based on consistent blood glucose level, six rats from each group were selected and other animals were excluded from further experiment; body weight, blood glucose and plasma insulin levels were checked at the end of experiment (day 14). Animals were killed on day 14. The experiment was performed after approval of the Committee for Laboratory Animal Use and Care of Daegu University. All procedures were executed in accordance with the Guide for the Care and Use of Laboratory Animals published by the National Institutes of Health.

### Quantitative real-time RT-PCR

Total RNA isolation kit (RNA-spin, iNtRON Biotechnology, Seongnam, Korea) was used to isolate total RNA from each group of tissue sample. Briefly, 1 microgram of RNA was used for conversion into cDNA using Maxime RT premix (iNtRON Biotechnology). The resulting cDNA was quantitatively analysed using Power SYBR Green (GE Healthcare, Warrington, UK) along with real-time RT-PCR (Stratagene 246 mx 3000p QPCR System, Agilent Technologies, Santa Clara, CA, USA). Primers used were rat *DJ1*-forward primer of base pairs 20 (5′-AACCATTCACTGGCCCGTTC-3′) and rat *DJ1*-reverse primer of base pairs 20 (5′-CTGGTTCACATGGTGGAGGG-3′). Transcript levels of the *DJ1* gene were normalized to the levels of *B2m* and *Ppib* using rat *B2m*-forward primer of base pairs 20 (5′-GTGTCTCAGTTCCACCCACC-3′) and rat *B2m*-reverse primer of base pairs 21 (5′-GGTGTGAATTCAGTGTGAGCC-3′), rat *Ppib-*forward primer of base pairs 20 (5′-GCCTCTCGGAGCGCAATATG-3′) and rat *Ppib*-reverse primer of base pairs 20 (5′-CTTATCGTTGGCCACGGAGG-3′).

### Immunoblot analysis

Liver tissue lysates were prepared with RIPA buffer (Sigma-Aldrich, St. Louis, MO, USA), homogenized, and centrifuged at 12,000 × g for 20 min. For immunoblot analysis, protein samples were prepared from six individual tissue samples per group, followed by quantification according to the Bradford method 21. Lysates were mixed with 2× sample buffer (50 mM Tris at pH 6.8, 2% SDS, 10% glycerol, 5% β-mercaptoethanol and 0.1% bromophenol blue) and heated for 5 min. at 95°C, followed by SDS-PAGE using an 8, 10, or 12% (w/v) polyacrylamide gel. After electrophoresis, proteins were transferred to a polyvinylidene difluoride membrane (Roche Diagnostics, Indianapolis, IN, USA) and then blocked for 1 hr in 5% skim milk prepared in TBS-T (Tris-buffered saline with Tween-20) buffer (10 mM Tris-HCl, 150 mM NaCl and 0.1% Tween 20). The membrane was rinsed three times consecutively with TBS-T buffer, followed by incubation for 1 hr with 1:1000 dilutions of primary monoclonal anti-β-actin, polyclonal anti-GRP78, anti-HSP70, anti-CA3, anti-CPS1 and anti-DJ1 antibodies (Santa Cruz Biotechnology, Santa Cruz, CA, USA) in TBS-T buffer containing 1% skim milk. After three washes, the membrane was incubated for 1 hr with horseradish peroxidase-conjugated anti-rabbit or anti-goat IgG secondary antibody (1:1000, AbFrontier, Seoul, Korea) in TBS-T buffer containing 1% skim milk. Membranes were then developed using enhanced chemiluminescence (Westzol, iNtRON Biotechnology). Chemiluminescence signal detection was performed with FUSION SOLO chemiluminescence and fluorescence imaging system (Vilber-Lourmat, Eberhardzell, Germany) and data were analysed using Kodak image analysis software (KODAK 1D; Estman Kodak, Rochester, NY, USA). Normalization was carried out using β-actin bands in each tissue.

### Immunofluorescence analysis

Tissue samples from livers of rats (*n* = 6) were dissected and stored in 10% neutral-buffered formalin. For immunofluorescence, formalin-fixed liver tissues were embedded in paraffin wax (Leica Microsystem, Wetzlar, Germany), after which tissues were sectioned to a thickness of 5 μm. To measure immunofluorescence, paraffin-embedded tissues were processed by serial dehydration and rehydration, followed by antigen retrieval using 10 mM sodium citrate solution. Sections were then washed with PBS-T three times, blocked with 5% BSA for 1 hr, incubated with polyclonal anti-DJ1 antibody (1:200 dilution; Santa Cruz Biotechnology) overnight at 4°C, washed three times with PBS-T and incubated again with rhodamine-conjugated anti-rabbit secondary antibody (1:1000) for 1 hr. Sections were counterstained with DAPI (Sigma-Aldrich) and mounted using commercially available mounting medium (Dako North America Inc., Carpinteria, CA, USA). For liver sections, double immunofluorescence was performed with mixture of anti-DJ1 antibody (1:200 dilution; Santa Cruz Biotechnology) and anti-CA3 antibody (1:200 dilution; Santa Cruz Biotechnology), then visualized after reaction with mixture of rhodamine-conjugated anti-rabbit IgG and FITC-conjugated donkey anti-goat antibody. Sections were counterstained with DAPI. Fluorescence images were captured using an Olympus IX51 inverted microscope (Olympus Co., Tokyo, Japan). Sections were observed at 400× magnification. Quantitative analysis of immunofluorescence for identifying localization and co-localization of DJ1 was carried out by analySIS FIVE Digital Imaging Solutions Software (Soft Imaging System GmbH, Johann-Krane-Weg, Münster, Germany).

### Immunoprecipitation and Co-immunoprecipitation

Immunoprecipitation (IP) was performed with a Pierce Direct IP kit (Thermo Fisher Scientific Inc. Rockford, IL, USA) as per the manufacturer's instructions. Specifically, 20 microgram of anti-DJ1 antibody (1:250; Santa Cruz Biotechnology) was used to precipitate the respective proteins. Rabbit IgG was used as a control. After elution of the proteins, all samples were separated by 10% SDS-PAGE, followed by immunoblotting with anti-DJ1 antibody (Santa Cruz Biotechnology). To identify DJ1-interacting partner proteins in the liver, Co-IP was performed with slight modifications as outlined by Tsai *et al*. 22. Tissue lysates (pooled sample of six rats per group) containing 300 mg of protein were incubated with 15 μl of anti-DJ1 antibody (Santa Cruz Biotechnology) at 4°C for 5 hrs on a rotary shaker, after which 20 μl of A/G PLUS agarose conjugate suspension (Santa Cruz Biotechnology) was added and allowed to mix at 4°C for 12 hrs on a rotary shaker. After centrifugation at 4000 × g for 5 min. at 4°C, the supernatant was collected and the beads were washed three times with RIPA buffer (Sigma-Aldrich). Then, 2× Laemmli buffer was added and boiled for 5 min. For the negative control sample, control rabbit and rat IgGs (Santa Cruz Biotechnology) were used. After Co-IP, all samples were separated by 10% SDS-PAGE and then silver-staining.

### Image acquisition and peptide mass fingerprinting

To identify partner proteins of DJ1, scrutinized gels were scanned on a UMAX Power Look 1120 (Maxium Technologies, Akron, OH, USA). Separated interacting partners were identified by protein mass fingerprinting (PMF). For this, excised protein bands were digested with trypsin (Promega, Madison, WI, USA) and mixed with α-cyano-4-hydroxycinnamic acid in 50% acetonitrile/0.1% TFA, followed by MALDI-TOF analysis (Microflex LRF 20, Bruker Daltonics, Billerica, MA, USA) as described by Fernandez *et al*. 23 using MASCOT (Mascot Sever 2.3) developed by Matrix science (http://www.matrixscience.com) as the search programme. Trypsin as the cleaving enzyme, a maximum of one missed cleavage, iodoacetamide (Cys) as a complete modification, oxidation (Met) as a partial modification, monoisotopic masses and a mass tolerance of ±0.1 D were used as parameters for the database search.

### Analysis of gene–gene interactions

To predict gene interactions between DJ1 and its interacting partner proteins identified in this study, we used a predictive web interface, GeneMANIA (http://www.genemania.org). This interface generates a list of genes with functional similarity based on the available genomics and proteomics database. There are two important parts of the GeneMANIA algorithm: a linear regression-based algorithm for calculating a single, composite functional association network from multiple networks derived from different proteomic or genomic data sources; and prediction of gene function 24,25.

### Validation of interaction between DJ1 and JNK1

Tissue lysates containing 300 mg of protein were incubated with anti-DJ1 antibody (1:250; Santa Cruz Biotechnology) or normal IgG mouse (1:250; Santa Cruz Biotechnology) at 4°C overnight on a rotary shaker. Protein A/G agarose beads were then added to the mixture and the tubes were centrifuged at 4000 × g at 4°C for 5 min. to precipitate the beads bound to the Ag-Ab complex. The beads were washed extensively and were centrifuged at 10,000 × g for 1 min., and all the liquid was thoroughly removed. SDS sample loading buffer (50 μl) containing 10% β-mercaptoethanol was then added to the agarose beads and heated at 95°C for 5 min. The denatured proteins were analysed by immunoblotting probing with monoclonal mouse anti-JNK1 antibody (1:1000; Santa Cruz Biotechnology). Proteins were visualized using enhanced chemiluminescence as described above.

### Statistical analysis

All experimental results are expressed as mean ± SD and compared by one-way anova using the Statistical Package of Social Science (SPSS, version 14.0K; Technologies, Chicago, IL, USA) program and Student's *t*-test. Statistical significances between male and female rats as well as controls and diabetic rats were expressed as either *P* < 0.05 or *P* < 0.01.

## Results

### Diabetic phenotype

**Figure 1:**
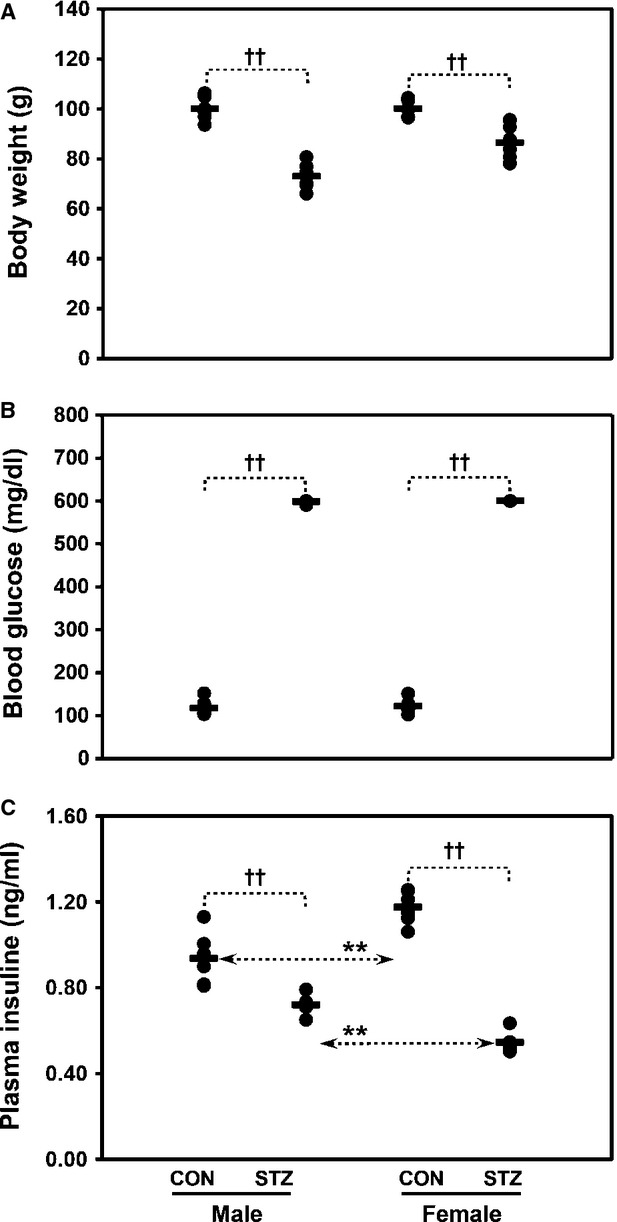
Total body weight (A), blood glucose levels (B), and plasma insulin levels (C) in healthy control (CON) and STZ-induced diabetic (STZ) rats of both genders upon completion of the experiment. One-way anova was used to calculate statistical significance between male and female rats, where *P*-value is *P < 0.05, **P < 0.01. Significance between controls and diabetic rats is depicted by ^†^*P* < 0.05, ^††^*P* < 0.01. Data represent mean ± SD (*n* = 6). Some data were adapted from Ref. 26 after modifications as a baseline data.

Prior to the experiment, rats were divided randomly into two groups of males (*n* = 24) and females (*n* = 24). To investigate the role of DJ1 in T1DM, we developed an experimental diabetic model using STZ (50 mg/kg body weight), as outlined in a previous study by our group 26, and performed biochemical as well as protein–protein interaction studies focused on DJ1 expression with respect to T1DM in livers of male and female rats. Total body weight, blood glucose and plasma insulin levels were measured at the end of the experiment (day 14) (Fig.1A–C, respectively). Two weeks after STZ treatment, male rats showed greater body weight reduction compared to their control counterparts than female rats (Fig.1A). Significant elevation of blood glucose levels in both genders indicated progression of diabetes (Fig.1B). As expected, significant decrease in insulin level was observed in both genders (Fig.1C). Remarkably, females were more prone to developing diabetes as a result of lower insulin secretion as compared to their male counterparts.

### Tissue-specific expression of DJ1

**Figure 2:**
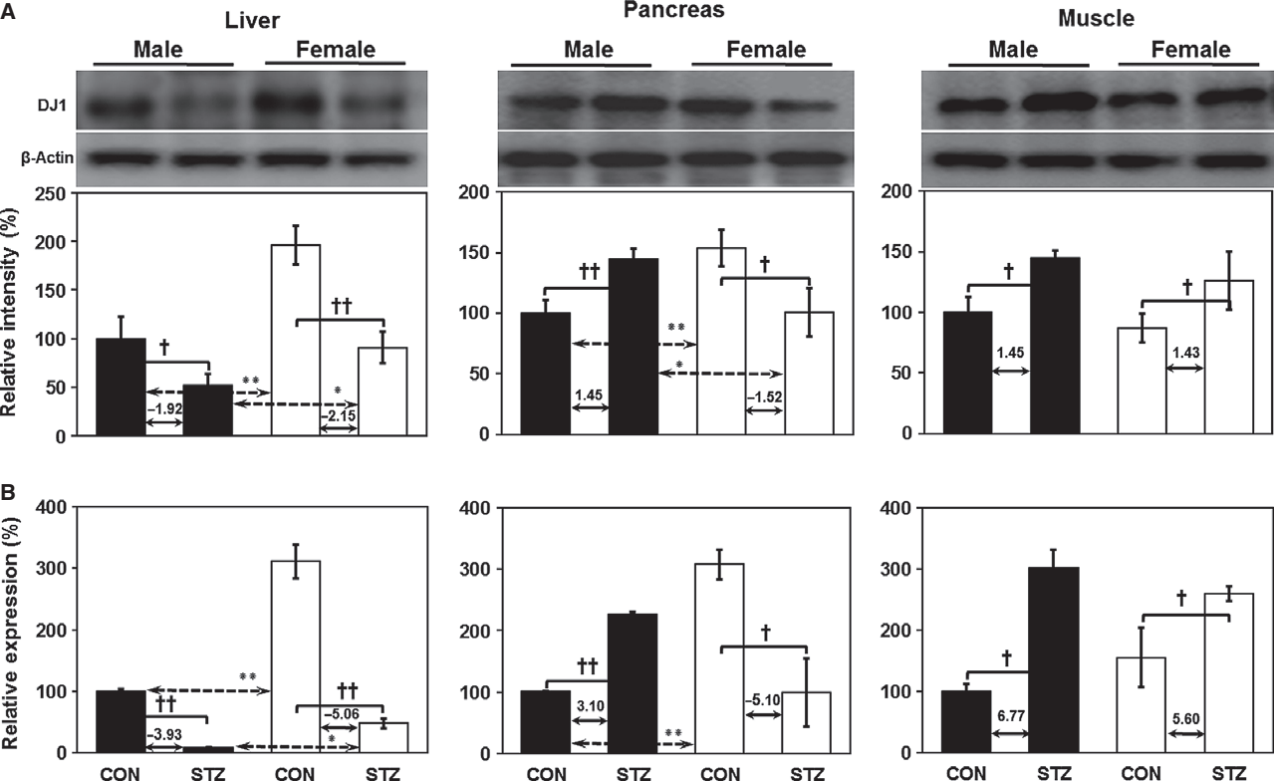
Tissue-specific relative expression of DJ1 in the liver, pancreas and skeletal muscle of both males and females (healthy male control *versus* diabetic male rats; healthy female control *versus* diabetic female rats) at the (A) protein and (B) mRNA levels (depicted values in graph indicate fold change in protein and gene expression, respectively). Band density was calculated using ImageMaster 2D software version 4.95. Relative intensity (%) values of proteins were normalized to β-actin levels, whereas mRNA levels were normalized to *B2m* and *Ppib*. One-way anova was used to calculate statistical significance between male and female rats, where *P*-value is *P < 0.05, **P < 0.01. Significance between controls and diabetic rats is depicted by ^†^*P* < 0.05, ^††^*P* < 0.01. Data represent mean ± SD (*n* = 6).

To understand the tissue-specific expression of DJ1 in three different metabolic tissues (liver, pancreas and skeletal muscle), we performed immunoblotting and real-time PCR analysis. Interestingly, STZ induction significantly reduced the protein as well as mRNA expression levels of DJ1 in the livers of both genders (Fig.2A and B, respectively). Gender-dependent expression of DJ1 was observed in the pancreas, as male rats showed elevated DJ1 expression while females exhibited remarkably reduced expression levels after STZ induction (Fig.2A and B). Conversely, in skeletal muscle, elevated protein and mRNA levels of DJ1 were observed in both genders (Fig.2A and B, respectively).

### Immunofluorescence analysis of DJ1

**Figure 3:**
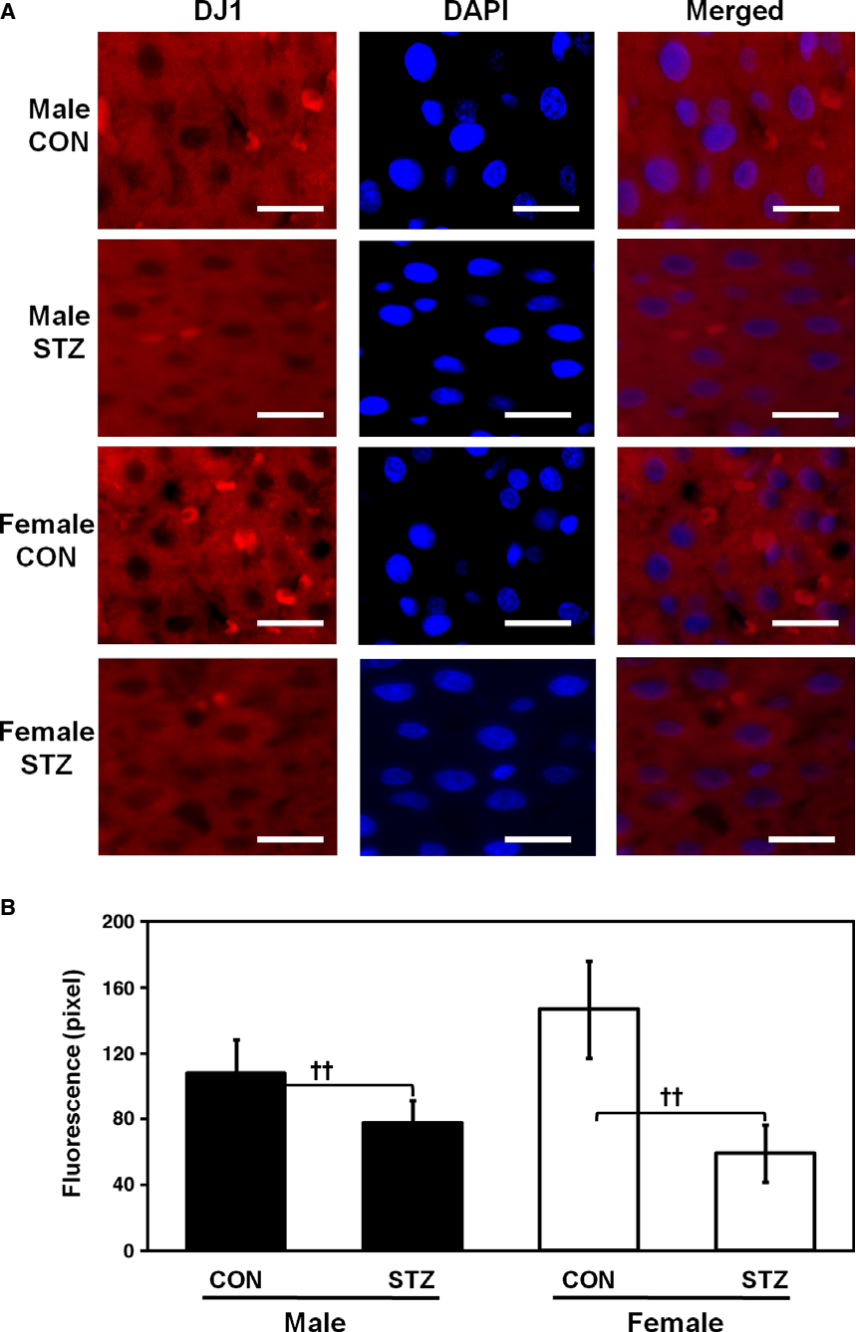
Immunofluorescence corroborated expression pattern of DJ1 by Western blotting and real-time PCR from the livers of CON as well as STZ-induced male and female rats. Liver sections from rats were immunostained with anti-DJ1 antibody and then visualized after reaction with rhodamine-conjugated anti-rabbit IgG by nuclear staining with DAPI. Sections were observed at 400 ×  magnification, scale bar represents 50 μM (A). Quantitative analysis of immunofluorescence (B). Student's *t*-test was used to calculate statistical significance between male and female rats, where *P*-value is *P < 0.05, **P < 0.01. Significance between controls and diabetic rats is depicted by ^†^*P* < 0.05, ^††^*P* < 0.01. Data represent mean ± SD (*n* = 6).

To investigate the location and expression pattern of DJ1 in the liver, histological characteristics were determined by immunostaining with anti-DJ1 antibody. Immunofluorescence results corroborated the expression patterns of DJ1 in livers of CON as well as STZ-induced male and female rats determined previously by Western blotting and real-time PCR. As shown in Figure3, which is a representative image of three independent experiments, the majority of DJ1 was present in the cytoplasm of hepatocytes. In addition, we observed cytoplasmic co-localization of DJ1 and CA3 by double immunofluorescence staining (Fig.7).

### Identification of partner proteins of DJ1

**Figure 4:**
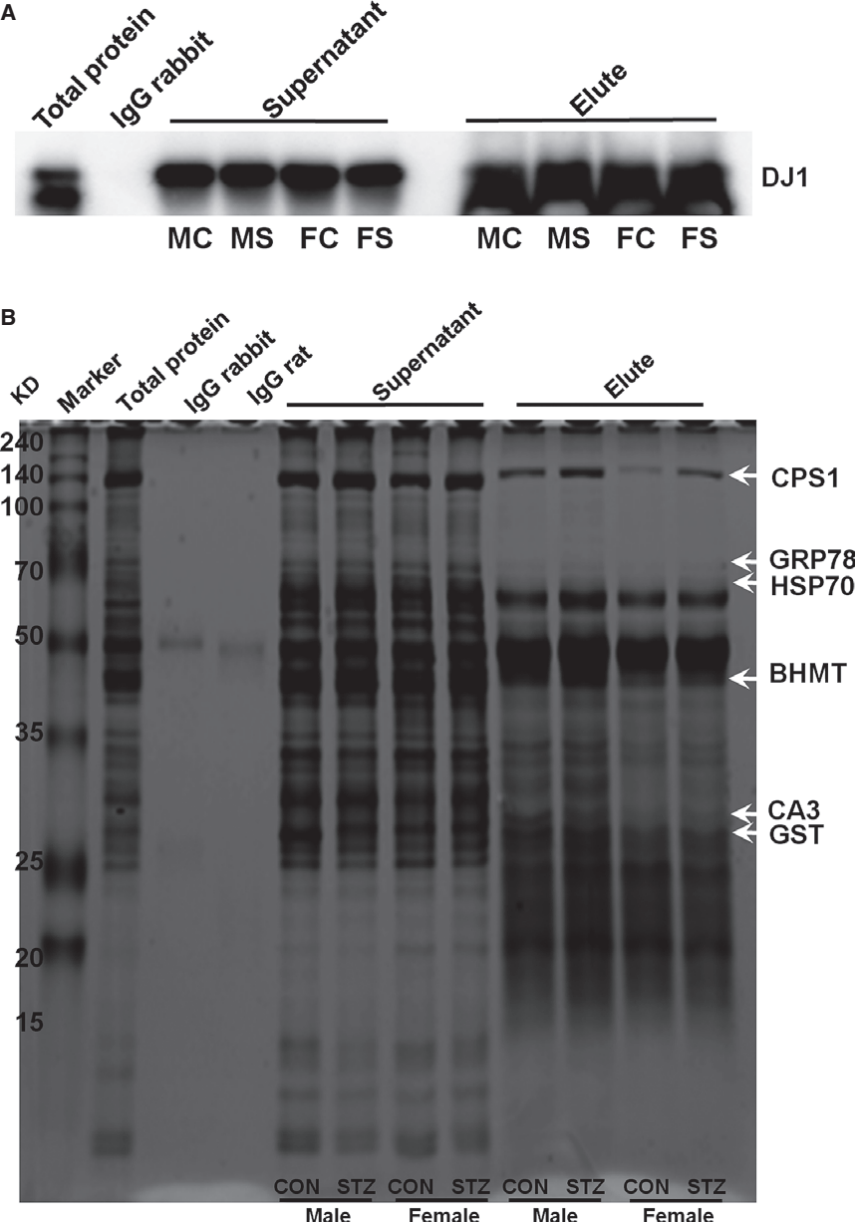
Representative immunoblot image of immunoprecipitated sample from the liver (A) (where, MC, male CON; MS, male STZ; FC, female CON; FS, female STZ). Representative silver-stained SDS-PAGE image of co-immunoprecipitated sample from the liver (B) (*n* = 6). Proteins were identified by PMF analysis and are indicated by arrows along with their abbreviated names.

**Figure 5:**
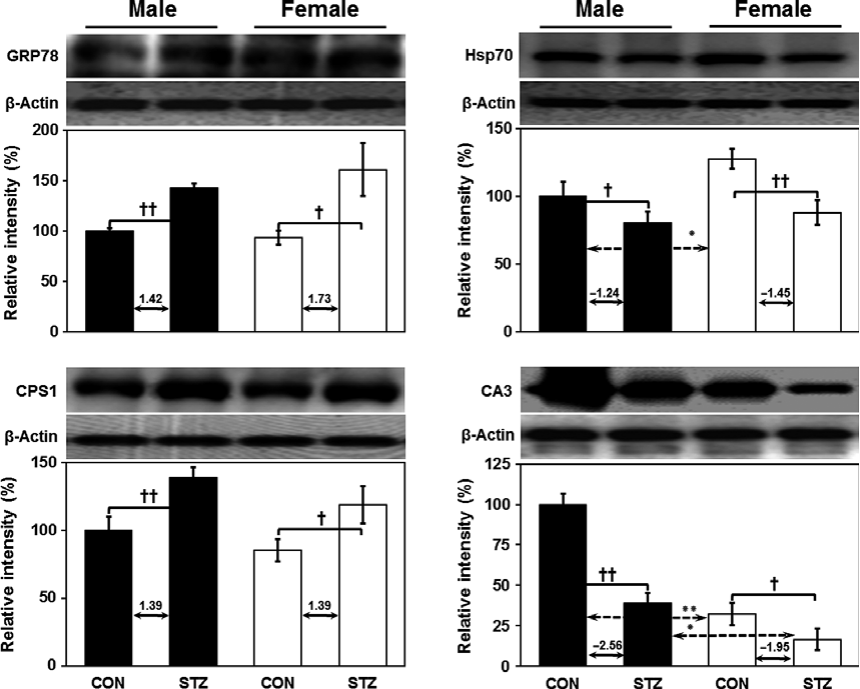
Validation of protein–protein interaction of DJ1 with 78 kD glucose-regulated protein (GRP78), stress-70 protein (Hsp70), carbamoyl-phosphate synthase (CPS1) and carbonic anhydrase 3 (CA3) in the liver by Western blotting. For immunoblotting, we used original pooled sample (*n* = 6). (Depicted values in graph indicate fold change in protein expression). Band density was calculated using ImageMaster 2D software version 4.95, and relative intensity (%) values of proteins were normalized to β-actin levels. One-way anova was used to calculate statistical significance between male and female rats, where *P*-value is *P < 0.05, **P < 0.01. Significance between controls and diabetic rats is depicted by ^†^*P* < 0.05, ^††^*P* < 0.01. Data represent mean ± SD.

Immunoprecipitation was used to isolate and concentrate DJ1 protein from sample containing thousands of different proteins. For this, anti-DJ1 antibody targeting the protein of interest was incubated with tissue extract to facilitate antibody binding to the protein in solution. The antibody/antigen complex was then pooled using protein A/G PLUS agarose beads, after which the protein of interest was isolated from the rest of the sample and confirmed by immunoblotting (Fig.4A). To identify interacting partners of DJ1 protein that are involved in the metabolic regulation of T1DM, we performed Co-IP of DJ1-associated proteins from rat liver samples. Proteins were separated by 10% SDS-PAGE, followed by silver-staining and PMF analysis. As shown in Figure4B, PMF analysis identified six important stress-related interacting protein partners, including 78 kD glucose-regulated protein (GRP78), stress 70 protein (Hsp70), carbonic anhydrase 3 (CA3), carbamoyl-phosphate synthase (CPS1), betaine-homocysteine S-methyl transferase (BHMT), and glutathione S-transferase (GST). Some of these interactions were further validated by direct immunoblot analysis (Fig.5).

### Prediction of gene functions

**Figure 6:**
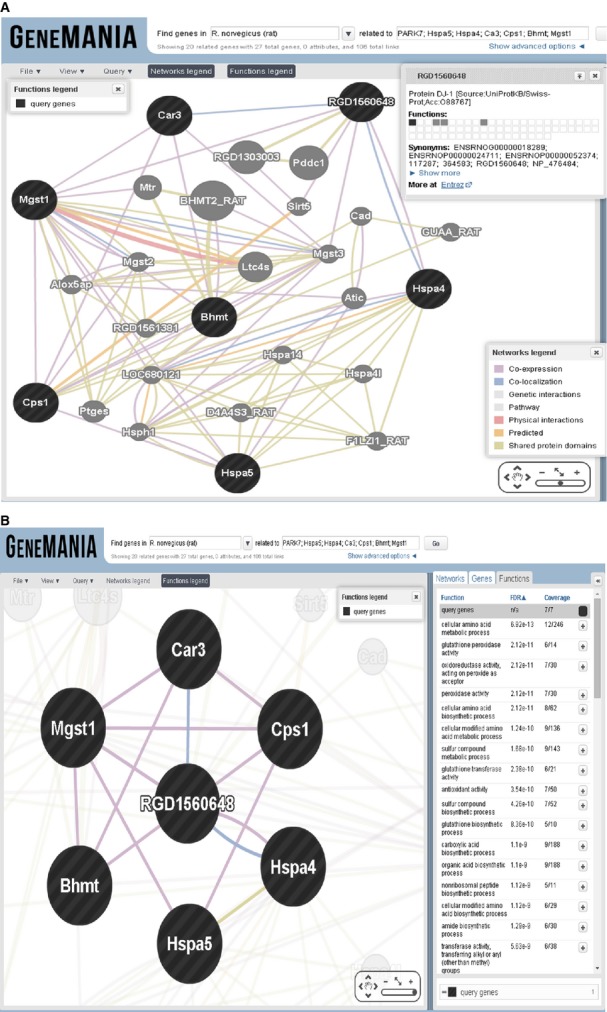
GeneMANIA showing the results of seven queries (encircled with black and bold) using the following advanced parameters: molecular function-based (A). GeneMANIA result window depicts close-up of interaction of only query genes. Extreme right panel depicts the functions of the given queries (B).

GeneMANIA was employed to determine the *in silico* gene interactions for proteins identified in the Co-IP experiment. Figure6A describes the interactions of seven query genes *viz*. *RGD1560648* (DJ1/PARK7), *Hspa5* (GRP78), *Hspa4* (HSP70), *Car3* (CA3), *Cps1* (CPS1), *Bhmt* (BHMT) and *Mgst1* (GST). To gain insight into genes of interest using GeneMANIA, we captured a screenshot of the close-up of only query genes by hiding all other interactions (Fig.6B). In addition, we predicted a possible interaction between DJ1 and JNK1 (Fig.8A). Function-based interaction of DJ1 with JNK1 *via* other interacting partners was presented in Fig.8B.

### Validation of interaction between DJ1 and JNK1

GeneMANIA search result showed that DJ1 interacts with JNK1. Therefore, to verify the possibility of such interaction, DJ1 was immunoprecipitated and the immunoblotting was performed for JNK1. It was observed that DJ1 was co-immunoprecipitated with JNK1 (Fig.9). This result suggests that JNK1 interacts with DJ1, indicating that DJ1 plays a JNK1- mediated important role in T1DM.

## Discussion

This study elucidates the gender-dependent expression of DJ1 in various metabolic tissues as well as its interactions with hepatic proteins after STZ induction. DJ1 protein plays a role in cell survival upon external insult of pancreatic β-cells as well as mouse pancreatic islets 27. Our earlier findings demonstrated that DJ1 is highly expressed in response to diet-induced oxidative stress in various metabolic tissues (liver, white adipose tissue, gastrocnemius and soleus muscle) of rats fed a HFD and treated with sex hormone 28. It has been suggested that DJ1 may play a potential role in STZ-induced T1DM metabolism.

The preliminary outcome of this study suggests that female rats are more vulnerable to diabetes as compared to their male counterparts. In this study, down-regulation of DJ1 protein as well as mRNA expression levels was observed in livers of both male and female rats after STZ induction (Fig.2). Similarly, down-regulation of DJ1 has been observed in hearts of male SD rats after STZ induction 20.

DJ1 helps reduce ROS in the mitochondria of insulin-secreting β-cells 14. In this study, gender-dependent expression of DJ1 was observed in the pancreas, as DJ1 was up-regulated in males but down-regulated in female rats after STZ treatment (Fig.2). In support of our findings, up-regulation of DJ1 has been reported in islets of HFD-induced hyperglycemic male mice 18. Conversely, elevated levels of DJ1 were found to be associated with antioxidant activity in skeletal muscle (Fig.2). To date, no gender dimorphism of DJ1 expression has been observed in skeletal muscle. Further, an earlier study also reported that DJ1 is located mainly in the cytoplasm and nucleus of hepatocytes 29.

Oxidative stress, endoplasmic reticulum (ER) stress, inflammatory cytokines, and other metabolic stresses play a major role in β-cell dysfunction 30,31. In this study, we identified important interacting partners of DJ1 in T1DM (Fig.4B). According to the results of predictive GeneMANIA analysis, *RGD1560648* (DJ1) was found to interact with other partners *via* peroxidase, oxidoreductase, antioxidant activity and other various metabolic and biosynthetic pathways (Fig.6A).

The most remarkable outcome of this study is the identification of several interacting partner proteins of DJ1 in hepatic tissue of diabetic rats. The first example is 78 kD glucose-regulated protein (GRP78), which is a key member of the molecular chaperone family in the lumen of the ER and is involved in the regulation of secretory proteins 32. Elevated levels of GRP78 in diabetic rats of both genders reflect the ability to overcome cellular stress generated in mitochondria during T1DM. The expression pattern of GRP78 in this study is in line with our previous findings 12 as well as the results of an independent study using male Wistar rats 33. As an opposing result, reduced level of GRP78 has been reported in livers of diabetic Zucker rats 32 as well as in *db/db* mice 34. Overexpression of GRP78 has been noted to protect against various metabolic and toxic stimuli 35,36. Several reports have suggested that ER stress is involved in pancreatic β-cell loss, insulin resistance and pathogenesis of diabetes 37,38. It is well-established that upon onset of ER stress, ER transmembrane receptors evoke the unfolded protein response to restore normal ER function 39. In this study, we found that reduced levels of DJ1 in livers of STZ-induced SD rats of both genders may lead to ER stress, as loss of DJ1 or reduced protein activity is known to generate ER stress 27. Taken together, coordinated reduction in DJ1 expression along with increased GRP78 expression contribute to the altered redox status of the ER and its chaperones in diabetic rats, which may lead to impaired protein modification and folding in diabetic liver tissue.

An important interacting protein partner identified in this study is heat shock protein 70 (HSP70). This protein is an important part of the cellular machinery for protein folding, saves cells from stress by re-folding of denatured proteins, maintains structural integrity and acts as a molecular chaperone 40,41. In hyperglycemia, HSP70 expression is highly tissue-specific in STZ-induced male SD rats 42 as well as type 2 diabetic monkeys 43. Reduced HSP70 levels have been reported in skeletal muscle of type 2 diabetic patients 44,45, livers of type 2 diabetic monkeys 43, and both liver and muscle tissues of STZ-induced male Wistar rats 41,46. Further, expression of HSP70 is reduced during diabetes, resulting in impaired cytoprotection in diabetic rats 41. In this study, we observed down-regulation of HSP70 in livers of both genders of rats after STZ induction (Fig.5). In support of our findings, reduced expression of HSP70 has been reported in brains of female albino rats 47. Recently, Chien *et al*. demonstrated that β-cell apoptosis is due to misfolding of the islet β-cell peptide hA (human amylin) into β-sheet-containing oligomers. They also specifically demonstrated that HSP70, GRP78/BiP, and HSP40/DnaJ all play roles in suppression of hA misfolding. Moreover, HSP70 and GRP78/BiP can detect and bind to misfolded hA oligomers, resulting in protection of hA against bulk misfolding and irreversible aggregation 48. Collectively, these results suggest combined/coordinated chaperon activity for DJ1, GRP78 and HSP70 in T1DM.

Predictive analysis of genes of interest using GeneMANIA confirmed co-expression, co-localization, and shared protein domain-based direct interactions among *RGD1560648* (DJ1), *Hspa4* (HSP70), and *Hspa5* (GRP78), which implies a coordinated response during T1DM. Li and coworkers have shown that DJ1 protein is associated and localized with several chaperones, and its protease activity was found to be facilitated by HSP70 in human 293T cells and mouse NIH3T3 cells 49. In support of our findings, an earlier study demonstrated that DJ1 may possess chaperone activity and abolish aggregation of proteins 50. Taken together, the association of DJ1 with HSP70 and GRP78 clearly indicates its role in post-translational modifications during ER stress in T1DM.

**Figure 7:**
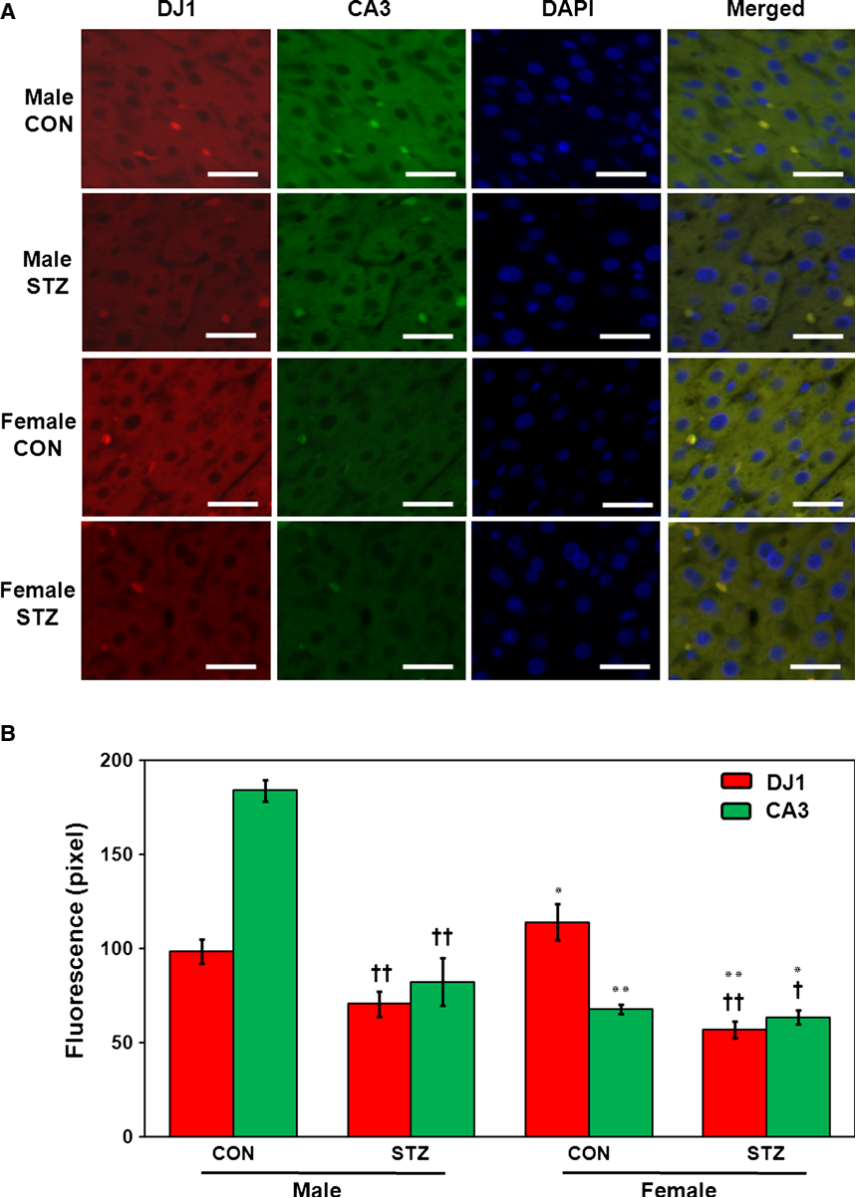
Co-localization of DJ1 and CA3 by double immunofluorescence. Double labelling with DJ1 (red) and CA3 (green) indicates that these markers are co-expressed in liver tissues. Further nuclei were stained with DAPI (blue). Sections were observed at 400 ×  magnification and scale bar represents 50 μM (A). Quantitative analysis of immunofluorescence (B). Student's *t*-test was used to calculate statistical significance between male and female rats, where *P*-value is *P < 0.05, **P < 0.01. Significance between controls and diabetic rats is depicted by ^†^*P* < 0.05, ^††^*P* < 0.01. Data represent mean ± SD (*n* = 6).

Another partner protein of DJ1 identified in this study is CA3, which participates in protecting cells from oxidative damage by scavenging oxygen radicals 51–53. Although its exact physiological role remains unclear, CA3 is known to possess efficient phosphatase activity 54. Interestingly, we found no sexual dimorphism in the expression of CA3, but we did detect higher expression in males as compared to female counterparts (Fig.5). In line with our findings, Kuhara *et al*. reported no sexual dimorphism along with 20% percent higher expression of CA3 in males as compared to female LEC rats 55. In contrast, elevated expression levels were reported in male Swiss albino rats after STZ induction 53. It has also been reported that lack of CA3 expression negatively affects mitochondrial ATP synthesis 56, and recent evidence has demonstrated reduced ATP synthesis in muscle tissue from T1DM patients 57. Prediction using GeneMANIA also confirmed lyase activity for CA3. Therefore, it can be concluded that reduction in CA3 expression plays an important role in T1DM development *via* ATP synthesis and contributes to the protection of proteins and cellular machinery from oxidative stress. Importantly, the prediction results using GeneMANIA also indicate that CA3 interacted directly with almost all identified partners except *Hspa4* and *Hspa5* (Fig.6). However, *Mgst1*- and *RGD1560648-*based interactions were observed in the case of *Hspa4* and *Hspa5*. Intensity of co-localization pattern was evaluated the relative levels of co-expression of CA3 to DJ1 in liver by double-immunofluorescence technique (Fig.7).

Betaine-homocysteine methyltransferase (BHMT), also an important partner protein of DJ1, catalyses the transfer of methyl groups from betaine to homocysteine to form dimethylglycine and methionine, and it is involved in the remethylation pathway 58. In addition, GeneMANIA analysis confirmed a role for *Bhmt* (BHMT) in various metabolic processes *viz*. amino acid biosynthesis and metabolism, transferase activity, carboxylic acid biosynthesis, sulphur compound biosynthesis, amine biosynthetic process, *etc*. (Fig.6A). Alteration of BHMT activity may be associated with phospholipid metabolism 58. It has been documented that elevation of BHMT activity and mRNA levels occurs in livers of diabetic male rats, and reduced levels of choline have been reported in female T1DM patients 58–60. Increased BHMT activity may be associated with increased choline requirements in diabetic rats 60. In addition, it was shown that increased BHMT expression during diabetes induces the production of phosphatidylcholine along with involvement of phosphatidyl ethanolamine methylation 58. Therefore, it is likely that DJ1 helps elevate the level of BHMT to promote recovery from T1DM *via* phosphatidylcholine.

We also identified CPS1, which plays a key role in protein and nitrogen metabolism, as a partner protein of DJ1. We found that CPS1 interacted with DJ1 and was up-regulated in both genders of rats after STZ induction (Fig.5). Previous findings from our laboratory support the expression pattern of CPS1 detailed in this study. Specifically, we previously reported a 3.2-fold increase in CPS1 protein levels in male SD rats after STZ treatment 61. Another study documented a 1.3-fold increase in CPS1 levels in STZ-induced male Wistar diabetic rats 62. As T1DM is characterized by oxidative stress and inflammation, CPS1 plays a pivotal role in diabetic nephropathy. Coordination of DJ1 with elevated CPS1 levels indicates an increasing demand for carbamoyl phosphate as well as increased involvement of CPS1 in the urea cycle. CPS1 plays a role in carboxylic acid-binding, amino acid-binding, as well as amide biosynthesis and metabolism (Fig.6A).

Glutathione *S*-transferase, one of the important markers of the antioxidant system, was also identified as an interacting protein of DJ1. Several reports have detailed the alteration of GST in hyperglycemia 12,63,64. GeneMANIA results also suggest that GST has antioxidant activity as well as glutathione peroxidase activity, oxidoreductase activity, peroxidase activity, glutathione transferase activity, peptide metabolic processing, glutathione binding activity, *etc*. (Fig.6A). A previous report from our laboratory demonstrated antioxidant activity for DJ1 along with other antioxidative proteins 28. Coordination of GST-DJ1 exerts a neuroprotective effect by reducing ROS-mediated neuronal injury in astrocytes of Wistar rats 65,66. Therefore, DJ1 may be involved in reduction in mitochondrial ROS in the liver *via* GST activity, leading to rapid detoxification of the tissue.

In addition, we predicted using GeneMANIA that DJ1 may regulate JNK1 for control of T1DM. Impaired insulin biosynthesis and secretion are known to lead to further insulin resistance 2,3. Generation of oxidative and ER stresses is a hallmark of diabetes, leading to activation of the JNK pathway 30,67. JNK isoforms have differential functions in insulin resistance 68, and genetic analysis has indicated that the effects of JNK1 on insulin resistance can be separated from the effects of JNK1 on obesity. Recent research has suggested that JNK1 has many functions related to cytokine production, lipid metabolism, and regulation of insulin resistance 69. Previous studies have also shown that JNK is required for insulin action in hepatocytes 69,70. It was reported that activation of JNK leads to suppression of insulin gene expression *via* oxidative stress. Therefore, suppression of the JNK pathway can protect β-cells from oxidative stress 30,71,72. Knockdown of JNK1 in the liver has been shown to significantly reduce blood glucose and insulin levels as well as enhance hepatic insulin signalling in diet-induced obese mice 73. Various attempts have been made to suppress the JNK pathway using synthetic as well as natural inhibitors 30,67,74. Therefore, it is likely that suppression of the JNK pathway is beneficial to overcoming diabetic complications.

**Figure 8:**
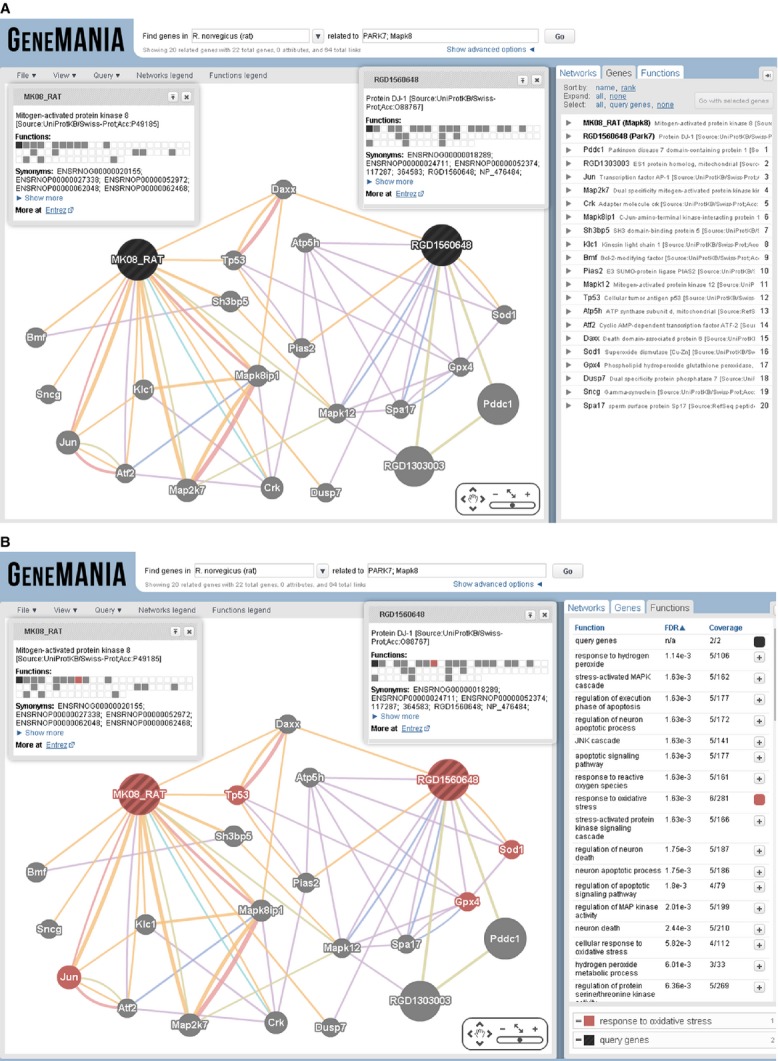
Prediction of role of DJ1 *via* JNK1 in T1DM (A). Screenshot of function-based relationship between DJ1 and JNK1 in GeneMANIA (B).

**Figure 9:**
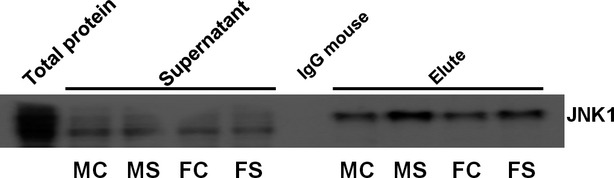
Validation of interaction between DJ1 and JNK1 by co-immunoprecipitaion, where DJ1 was immunoprecipitated and the immunoblotting was performed for JNK1 (*n* = 6).

In this study, using GeneMANIA predictive analysis, we found that *RGD1560648* (DJ1) participates in *Daxx*- and *Dusp7*-mediated interactions with *MapK8* (JNK1; Fig.8A and B). In line with our prediction results, Junn *et al*. demonstrated that DJ1 is a potent inhibitor of the Daxx/ASK1 cell-death signalling pathway in Parkinson's disease and is thus involved in cell survival 75. Moreover, in terms of a pathway ‘response to oxidative stress’, we were able to identify the link between *RGD1560648* (DJ1) and *Mapk8/MK08_Rat* (JNK1) (function-based relationship between DJ1 and JNK1, see Fig.8B), which allowed us to predict the association of DJ1 with JNK1 in T1DM, and it was further validated by co-immunoprecipitation (Co-IP; Fig.9).

Earlier studies have shown that DJ1 protects against UV-induced cell death through suppression of the JNK signalling pathway and that MEKK1 is the direct target of DJ1 76. These studies suggest that DJ1 plays a role in cell survival by suppressing the JNK pathway after UV induction. Similarly, DJ1 may promote β-cell protection/survival by suppressing the JNK pathway. We found *in silico* as well as physical interaction between DJ1 and JNK1 by GeneMANIA and Co-IP, respectively. However, further studies are required to directly test interactions between DJ1 and JNK1 in T1DM.

In conclusion, our data describe the involvement of DJ1 with several proteins from various metabolic pathways. However, further detailed studies are needed to understand the detailed mechanisms of the above-mentioned partner proteins in T1DM. Nonetheless, we anticipate that the current data will provide insights into the aetiology of T1DM.
